# Influence of social and meteorological factors on hand, foot, and mouth disease in Sichuan Province

**DOI:** 10.1186/s12889-023-15699-4

**Published:** 2023-05-10

**Authors:** Xiaohong Jiang, Yue Ma, Qiang Lv, Yaqiong Liu, Tao Zhang, Fei Yin, Tiejun Shui

**Affiliations:** 1 West China School of Public Health and West China Fourth Hospital, Sichuan University, Chengdu, Sichuan China; 2 Sichuan Center for Disease Control and Prevention, Chengdu, Sichuan China; 3 Yunnan Center for Disease Control and Prevention, Kunming, Yunnan China

**Keywords:** HFMD, Social factors, Relative humidity, Bayesian spatiotemporal model, Distributed lag nonlinear model

## Abstract

**Background:**

Hand, foot and mouth disease (HFMD) caused by a variety of enteroviruses remains a major public health problem in China. Previous studies have found that social factors may contribute to the inconsistency of the relationship patterns between meteorological factors and HFMD, but the conclusions are inconsistent. The influence of social factors on the association between meteorology and HFMD is still less well understood. We aimed to analyze whether social factors affected the effect of meteorological factors on HFMD in Sichuan Province.

**Method:**

We collected daily data on HFMD, meteorological factors and social factors in Sichuan Province from 2011 to 2017. First, we used a Bayesian spatiotemporal model combined with a distributed lag nonlinear model to evaluate the exposure-lag-response association between meteorological factors and HFMD. Second, by constructing the interaction of meteorological factors and social factors in the above model, the changes in the relative risk (RR) under different levels of social factors were evaluated.

**Results:**

The cumulative exposure curves for average temperature, relative humidity, and HFMD were shaped like an inverted “V” and a “U” shape. As the average temperature increased, the RR increased and peaked at 19 °C (RR 1.020 [95% confidence interval CI 1.004–1.050]). The urbanization rate, per capita gross domestic product (GDP), population density, birth rate, number of beds in health care centers and number of kindergartens interacted with relative humidity. With the increase in social factors, the correlation curve between relative humidity and HFMD changed from an “S” shape to a “U” shape.

**Conclusions:**

Relative humidity and average temperature increased the risk of HFMD within a certain range, and social factors enhanced the impact of high relative humidity. These results could provide insights into the combined role of environmental factors in HFMD and useful information for regional interventions.

**Supplementary Information:**

The online version contains supplementary material available at 10.1186/s12889-023-15699-4.

## Background

Hand, foot, and mouth disease (HFMD) is a common infectious disease caused worldwide by a variety of enteroviruses [1]. People may become infected with HFMD by contact with feces, respiratory secretions and herpes fluid of infected individuals [2], which have strong infectivity and are difficult to manage [3]. In recent decades, HFMD has mainly been prevalent in East and Southeast Asia. In China, approximately 1–3 million HFMD cases are reported every year [4]. Although clinical manifestations are usually self-limiting in 7–10 days for most patients with HFMD, some may develop severe symptoms, such as encephalomyelitis, respiratory tract infection and myocarditis, within days [5, 6]. However, the HFMD vaccine licensed in 2016 against enterovirus 71 (EV71) cannot effectively immunize against other strains [7]. Moreover, the proportion of dominant strains EV71 and Coxsackievirus A16 (Cox A16) in HFMD pathogens gradually decreased, and the proportion of other enteroviruses gradually increased [8, 9]. Therefore, HFMD is a major public health problem in China. To prevent and control this disease, we still need to explore its epidemiological characteristics and influencing factors.

Previous studies have found that HFMD is characterized by seasonal variation, spatial aggregation and spatial heterogeneity [10-13], which are related to meteorological factors and local social factors, including demographic and socioeconomic variables (population density, urbanization rate, per capita gross domestic product (GDP)), and health resources [14-16]. Social factors may influence not only the spatial distribution of HFMD but also the relationship between meteorological factors and HFMD in different regions [17]. Some studies have explored the influence of social factors on the association between HFMD and meteorological factors. However, the conclusions are inconsistent [18-22]. A study from China [18] found a strong interaction between temperature, humidity and per capita GDP, whereas studies from Guangdong [19] and multiple cities nationwide in mainland China [20, 21] did not find that per capita GDP modified the association between humidity, temperature and HFMD. The modification of hospital beds was found in another multicity study on humidity in mainland China [21] but was not significant in a study from the Sichuan Basin [22]. Whether social factors influence the effect of meteorological factors on HFMD remains unclear.

In addition, the above studies mainly used a two-stage distributed lag nonlinear model (DLNM) to analyze the explanatory ability of city-specific indicators on the heterogeneity of the association between meteorological factors and HFMD. Due to the features of DLNMs, the above studies could not consider the spatial autocorrelation of HFMD found in other studies [23]. At present, no study has analyzed the influence of social factors on the relationship between HFMD and meteorological factors based on the comprehensive consideration of the space–time distribution of HFMD. Therefore, more relevant studies are still needed, which is not only conducive to a deeper understanding of the role of the environment in the spread of HFMD but also to provide information for formulating appropriate local intervention strategies.

Sichuan Province is one of the provinces with a serious HFMD problem in China. Since 2008, the reported incidence of HFMD has increased and reached 71.59/100,000 in 2017. Moreover, the annual average reported case fatality rate from 2008 to 2017 reached 42.9/100,000, which ranked second in China [24]. In addition, economic development, demographics, and health services vary widely among different cities, which is convenient for exploring whether social factors have an impact on the association between meteorology and HFMD. Therefore, considering the spatial autocorrelation of HFMD, we adopted a Bayesian spatiotemporal model combined with a DLNM to fit nonlinear lag associations between meteorological factors and HFMD in Sichuan Province and to further analyze whether these associations changed at different levels of social factors. Our findings are helpful for understanding the comprehensive effects of social and meteorological factors on HFMD and provide information for implementing appropriate local intervention strategies.

## Methods

### Study region

**Fig. 1:**
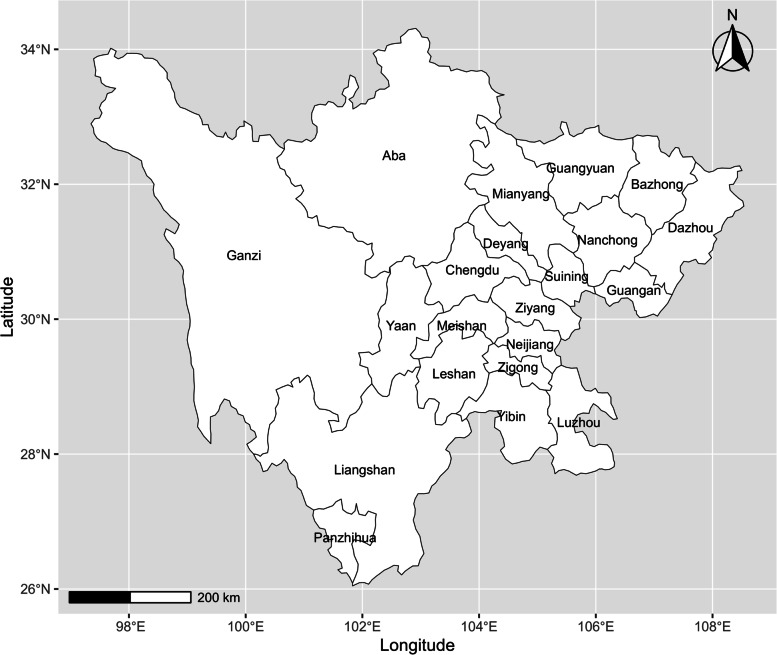
Map of cities in Sichuan Province (The base map is from the resource and environment science and data center)

Located between latitudes 26°03’-34°19’ north and longitudes 92°21’-108°12’ east, Sichuan covers an area of 486,000 square kilometers in southwestern China. It comprises 21 prefecture-level cities, mainly concentrated in the eastern region, and the scale of each city is considerably different (Fig. 1). The population, industry, technology, information and talent of Sichuan Province are highly concentrated in the capital city, more than in other cities.

### Data sources

The daily HFMD data in 21 prefectures in Sichuan Province from 2011 to 2017 came from the infectious disease reporting information management system of the Chinese Center for Disease Control and Prevention. Meteorological factors, including average temperature, relative humidity, sunshine hours, rainfall and wind speed, were derived from the daily monitoring data of 41 monitoring stations in Sichuan Province from 2011 to 2017 (Additional file 1: Fig. S1), obtained through the China Meteorological Data Network [25], and the Kriging interpolation method was used to calculate the daily data of meteorological variables for 21 prefecture-level cities [26].

Social factors were selected according to whether they were related to the transmission route and susceptible population of HFMD, as well as the availability of data [27]. The urbanization rate, per capita GDP, population density, birth rate, number of beds in health care centers and number of kindergartens were selected for analysis. Socioeconomic data were obtained from the Sichuan Statistical Yearbooks.

### Statistical analysis

**Fig. 2:**
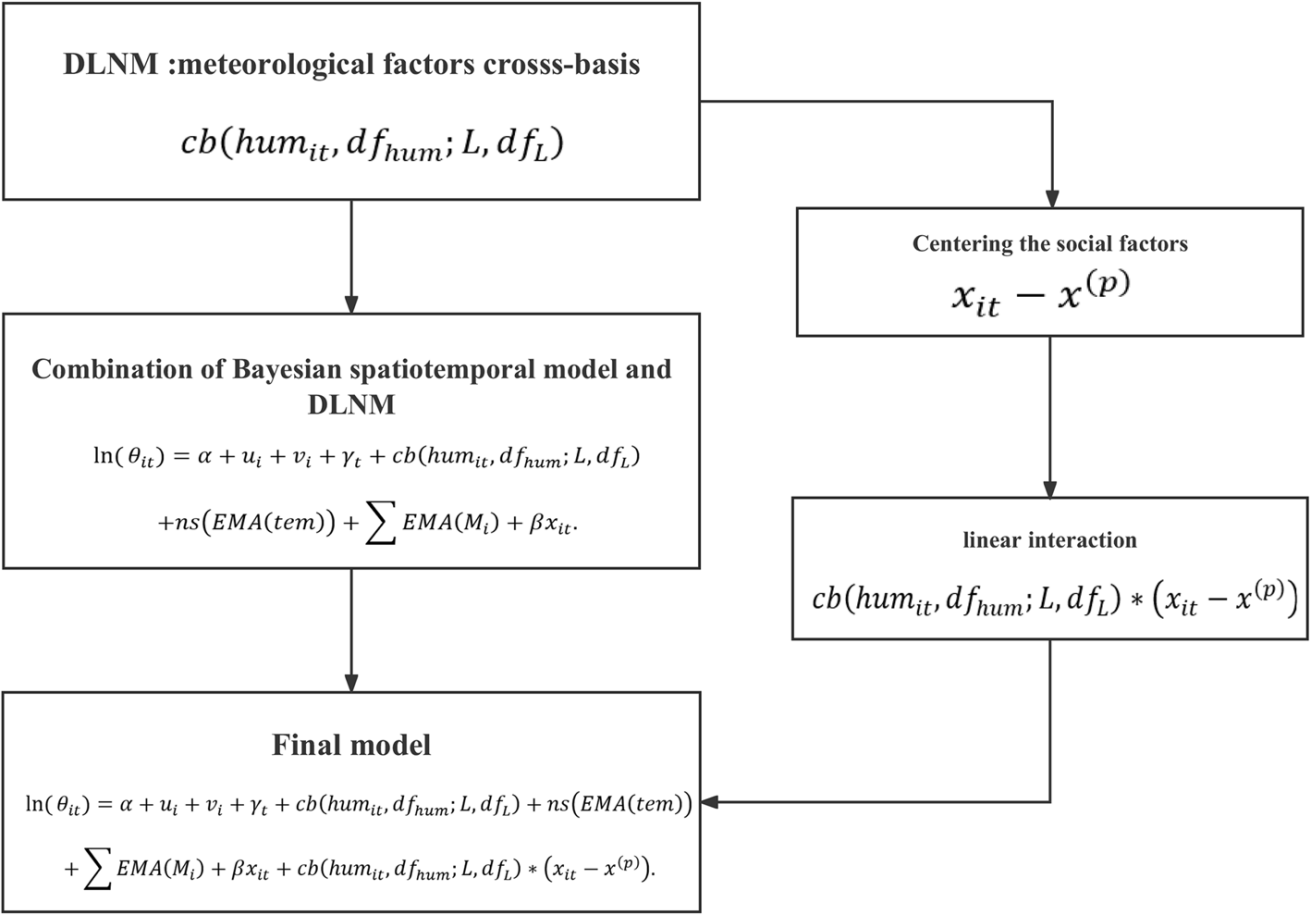
Model flowchart

We employed a Bayesian spatiotemporal model combined with a DLNM to investigate the influence of social factors on the relationship between meteorological factors and HFMD. First, the DLNM was used to construct the cross-basis functions of meteorological factors and then we incorporated them into the Bayesian spatiotemporal model to explore nonlinear-lag-response associations between meteorological factors and HFMD. Finally, to understand variations in the relationship between HFMD and meteorological factors by the levels of social factors, we added a linear interaction term between the cross-basis functions of meteorological factors and social factors to the Bayesian spatiotemporal model, in which different percentiles of social factors (90th, 50th, 10th) represented different levels of social factors. The flow chart was shown in Fig. 2.

### Distributed lag nonlinear models

Distributed lag nonlinear models based on the cross-basis function were used to explore the nonlinear and delayed associations between HFMD and meteorological factors. According to previous studies and the incubation period of HFMD, we assessed the delayed effects of meteorological factors on HFMD to be 14 days [28] and constructed meteorological factors cross-basis functions. To flexibly fit the complex association between meteorological factors and HFMD, natural cubic spline functions were used for both exposure and lag dimensions of the cross-basis functions. Based on the above cross-basis functions and centering the social factors on different percentiles (90th, 50th, 10th), the linear interaction between the meteorological factor cross-basis and social factors was obtained by multiplying the cross-basis functions with social factors.

### Combination of the Bayesian spatiotemporal model and DLNM

A Bayesian spatiotemporal model was constructed with the daily reported counts of HFMD for all 21 prefecture-level cities in Sichuan from January 2011 to December 2017 as the dependent variable. HFMD counts were assumed to have a negative binomial distribution to account for overdispersion. The spatiotemporal random effect was an independent variable that included the city-level daily autocorrelated random effect and year-specific city-level spatial random effect, accounting for seasonality, unobserved and unmeasured sources of variation and spatial dependency structure, respectively. We added the cross-basis function of meteorological factors and their linear interaction term with social factors to the Bayesian spatiotemporal model to construct the final model [29, 30]. The cross-basis function of meteorological factors in the final model represented the exposure-lag-response relationship corresponding to the centralized value of the social factor because the linear interaction term is 0 for that value. Taking the relative humidity as an example, the final formula is as follows:

1 $${y}_{it}\sim \text{NegBin }\left({E}_{it}{\theta }_{it},k\right)$$

where ${y}_{it}$, ${E}_{it}$ and ${\theta }_{it}$ are the number of reported and expected HFMD cases and relative risk (RR) on day $t (t=\mathrm{1,2},...,2557)$ in city $i (i=\mathrm{1,2},...,21)$, respectively; $k$ is the scale parameter.

2 $$\mathrm{ln}({\theta }_{it})=\alpha +{u}_{i}+{v}_{i}+{\gamma }_{t}+cb\left({hum}_{it},d{f}_{hum};L,d{f}_{L}\right)+ns\left(EMA\left(tem\right)\right)+\sum EMA\left({M}_{i}\right)+\beta {x}_{it}+cb\left({hum}_{it},d{f}_{hum};L,d{f}_{L}\right)*\left({x}_{it}-{x}^{\left(p\right)}\right).$$

α is the intercept; ${u}_{i}$ and ${v}_{i}$ are the spatially structured and unstructured components, respectively; and ${\gamma }_{t}$ is the temporally structured effect. The spatial random effect adopted a modified Besag-York-Mollie (BYM) model, and the temporally structured effect used a first-order random walk. The hyperprior parameters of spatiotemporal random effects were all based on penalized complexity (PC) priors; the accuracy was $\tau =1/{\sigma }^{2}$, $Pr\left(1/\sqrt{\tau }>0.5\right)=0.01$ [31]; $cb\left({hum}_{it},d{f}_{hum};L,d{f}_{L}\right)$ is the relative humidity cross-basis function with 4 degrees of freedom (df) in the exposure–response dimension and 4 df in the lag-response dimension; $L$, the maximum lag period, is 14 days;$EMA\left({M}_{i}\right)$ presents an exponential moving average of meteorological confounders ${M}_{i}$ with the same lag range as relative humidity; and $cb\left({hum}_{it},d{f}_{hum};L,d{f}_{L}\right)*\left({x}_{it}-{x}^{(p)}\right)$ is the linear interaction term between the relative humidity DLNM and social factor $x$. When ${x}_{it}$ is equal to ${x}^{(p)}$, the main effect $cb\left({hum}_{it},d{f}_{hum};L,d{f}_{L}\right)$ represents the exposure-lag-response association between HFMD and relative humidity corresponding to the ${x}^{(p)}$ level.

We mainly investigated the influence of relative humidity and average temperature on HFMD. The relative risk and 95% confidence interval (CI) were calculated using the median as a reference value. As the parameter specification and forms of meteorological confounders may influence the effect estimation, we conducted sensitivity analyses (Additional file 1: Figs. S2, S3, S4, S5, and S6.), including 1) varying df (3–7) in the cross-basis function for temperature and relative humidity and (2) changing the form of meteorological confounders (nonlinear and linear forms). Average temperature and relative humidity were included in nonlinear form, while rainfall, sunshine and wind speed were included in linear form. The parameters of the model were estimated utilizing the integrated nested Laplace approximation (INLA). The “INLA” and “dlnm” packages in R 4.0.3 were used to conduct all the analyses.

## Results

### Descriptive analysis

**Fig. 3:**
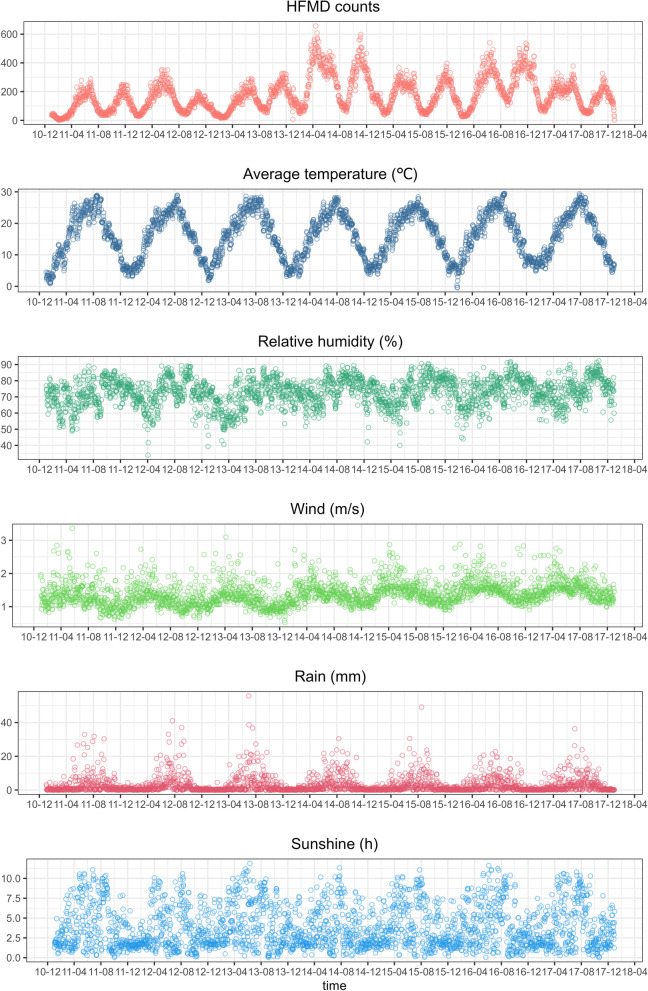
Temporal trends in HFMD and meteorological factors in Sichuan, 2011–2017

From January 1, 2011, to December 31, 2017, a total of 456,656 HFMD cases were reported in Sichuan Province, including 271,351 male cases and 185,305 female cases, with a sex ratio of 1.46. Table 1 describes the HFMD counts and meteorological and social factor data. The average daily HFMD cases in Sichuan Province were 178.6 and the daily average temperature and relative humidity were 16.2 °C and 72.6%, respectively. Social factors were annual averages from 2011 to 2017, as summarized in detail in Table 1. Both meteorological factors and HFMD showed seasonal patterns. HFMD showed a bimodal pattern, increasing from April to June and from October to December each year (Fig. 3). Except for population density, social factors such as urbanization rate, per capita GDP, birth rate, and the number of hospital beds and kindergartens gradually increased during the study. Population density showed the opposite trend, first declining and then fluctuating (Additional file 1: Figs. S7 and S8, Table S1).

**Table 1:** Statistical summary of daily HFMD counts and meteorological and social variables for Sichuan Province from 2011 to 2017

Variables	Mean ± SD	Min	*P*_25_	Median	*P*_75_	Max
Daily HFMD counts	178.6 ± 114.2	2.0	86.0	161.0	243.0	657.0
Meteorological factors						
Average temperature (℃)	16.2 ± 8.2	-16.2	9.7	16.7	22.7	35.8
Relative humidity (%)	72.6 ± 14.2	10.2	65.0	74.5	83.0	101.3
Wind speed (m/s)	1.4 ± 0.6	0.0	1.0	1.3	1.7	5.7
Rainfall (mm)	2.8 ± 8.2	0.0	0.0	0.1	1.9	240.3
Sunshine (h)	3.8 ± 3.7	0.0	0.1	2.7	7.1	12.8
Socioeconomic factors						
Per capita GDP (WYuan)	3.3 ± 1.5	1.0	2.3	2.9	3.7	9.3
Urbanization rate (%)	43.0 ± 9.7	22.4	37.2	41.9	46.5	71.9
Birth rate (‰)	9.7 ± 1.5	7.1	8.7	9.4	10.4	19.3
Population density (people per sq. km)	379.0 ± 270.8	7.2	166.5	343.0	535.0	1209.5
Number of beds in health care centers (10,000)	2.2 ± 2.1	0.3	1.2	1.8	2.2	13.5
Number of kindergartens	574.7 ± 395.8	61.0	300.0	467.0	720.0	2366.0

**Fig. 4:**
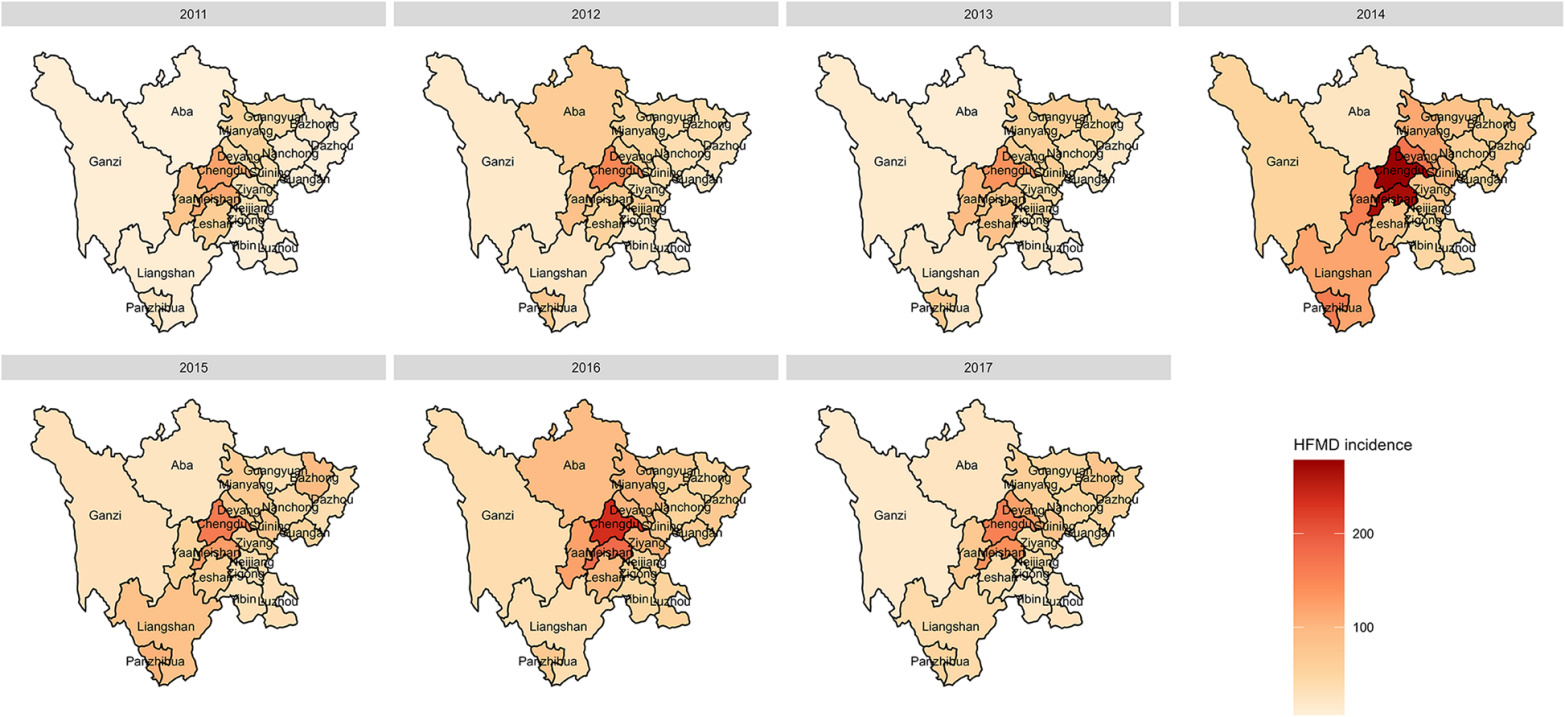
Annual prefecture-level HFMD incidence (1/100,000) from 2011 to 2017 in Sichuan (The base map is from the resource and environment science and data center)

**Fig. 5:**
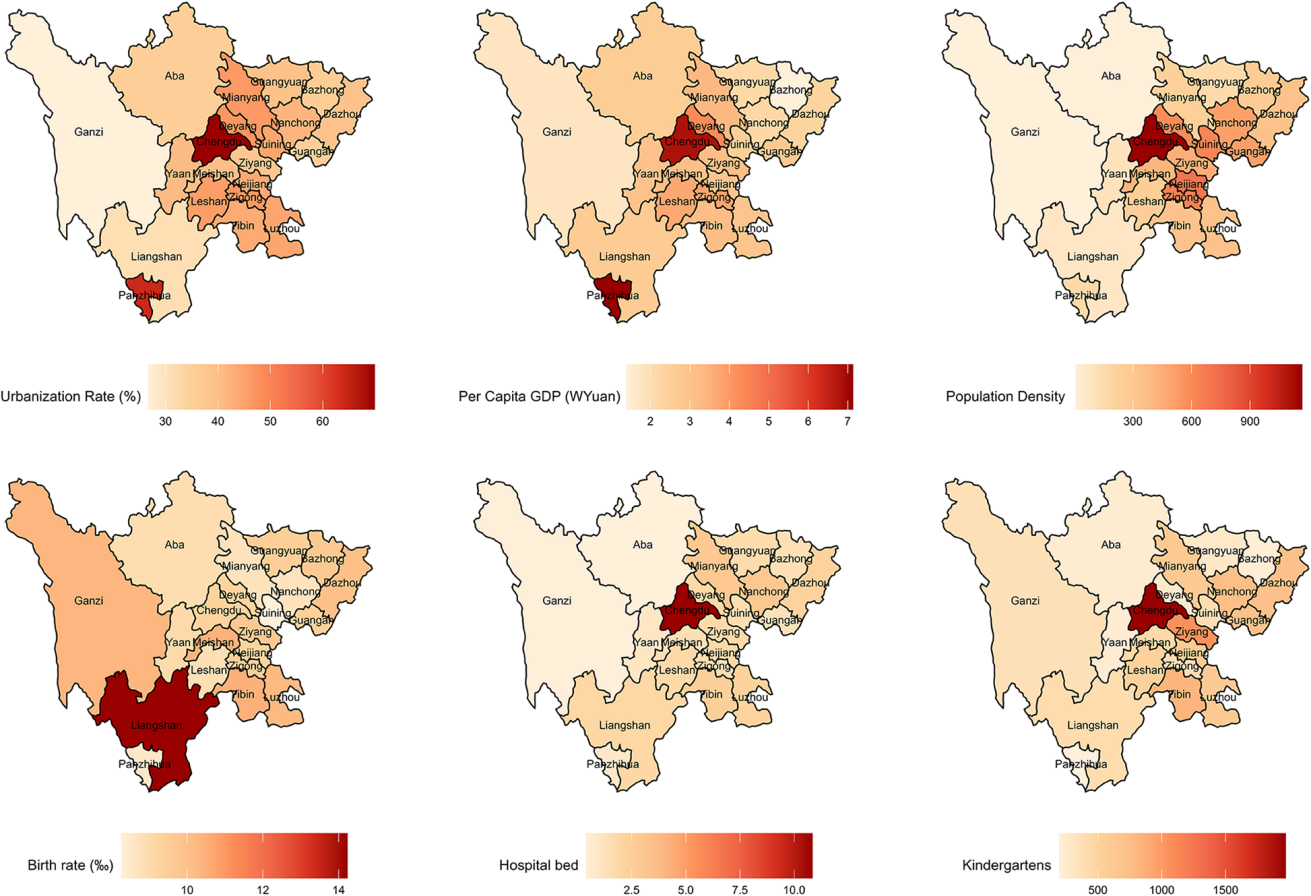
The average annual social factors in Sichuan, 2011–2017 (The base map is from the resource and environment science and data center)

The spatial distribution of the incidence of HFMD in Sichuan from 2011 to 2017 is shown in Fig. 4. The incidence of HFMD varied greatly among prefecture-level cities every year, and the areas with a high incidence were mainly in Chengdu and its surrounding areas and Panzhihua city. Except for 2014, the incidence of HFMD in Chengdu ranked first in Sichuan Province. Figure 5 shows the average annual social factors in the prefecture-level cities in Sichuan Province. The spatial distribution characteristics of the urbanization rate, per capita GDP, population density, number of beds in health care centers and number of kindergartens were similar to those of HFMD. Chengdu and surrounding prefecture-level cities had significantly higher average social factor levels than other cities. In addition, the urbanization rate and per capita GDP of Panzhihua were also higher than those of its surrounding areas. The spatial distribution of the birth rate was opposite to that of other social factors. The areas with high birth rates were mainly concentrated in the southern and western regions of Sichuan Province, and the birth rate was lower in Chengdu and its surrounding areas.

### The associations of meteorological factors with HFMD

**Fig. 6:**
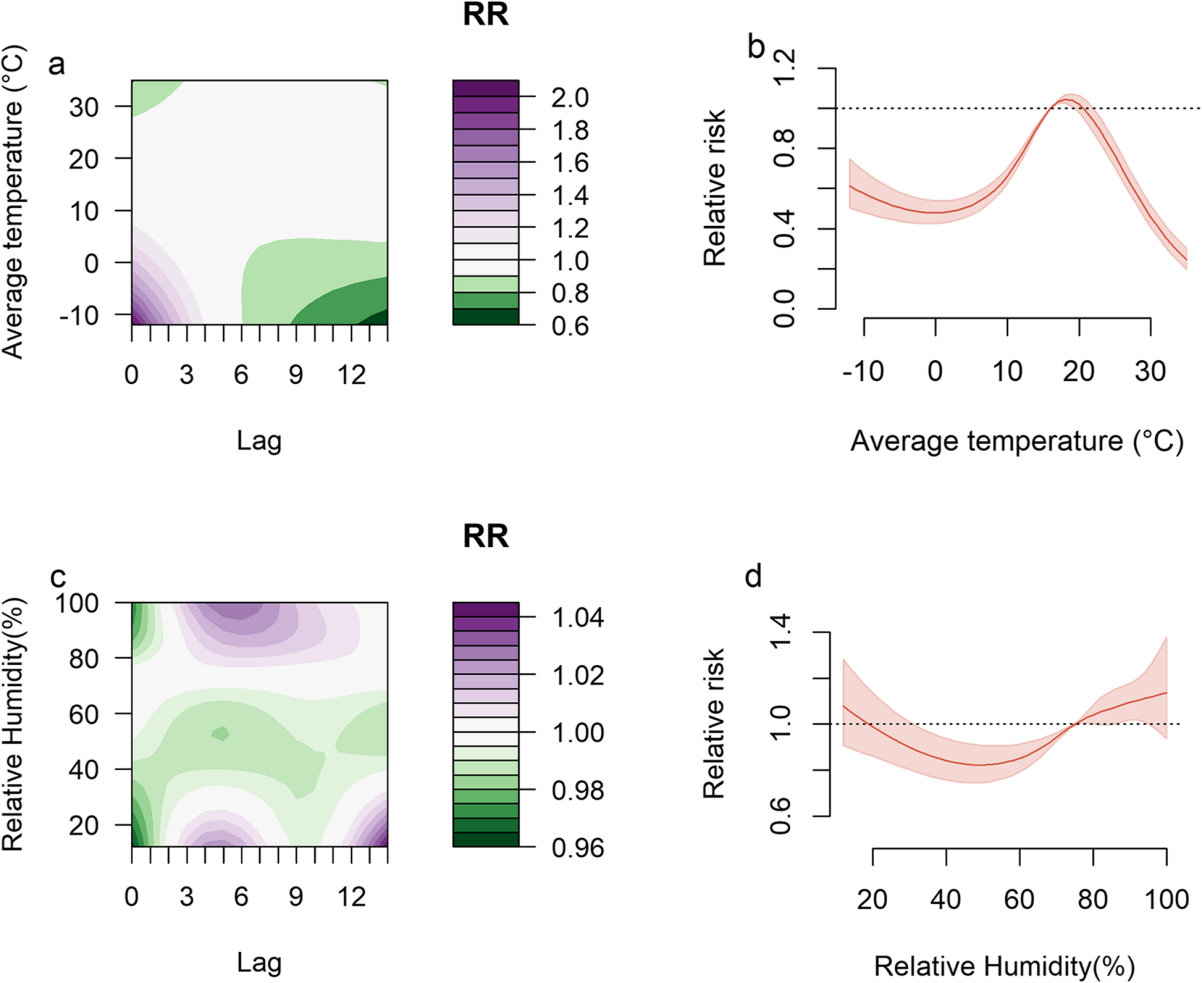
The relationship between HFMD and meteorological factors. **a** Contour plot of average temperature relative to the overall median temperature (17 °C). **b** HFMD cumulative exposure–response association for average temperature relative to the overall median temperature (17 °C). **c** Contour plot of relative humidity relative to the overall median relative humidity (75%). **d** HFMD cumulative exposure–response association for relative humidity relative to the overall median relative humidity (75%)

The results of the average temperature model constructed in the second step are shown in Fig. 6. When the daily average temperature was lower than 7 °C, the risk of HFMD was highest on that day and decreased gradually over time, reaching the lowest on lag day 14 (Fig. 6a). Figure 6b shows the cumulative RR curve of the average temperature, which is similar to an inverted "V" shape. When the daily average temperature was lower than 19 °C, the cumulative RR of HFMD first decreased and then increased with increasing average temperature and peaked at 19 °C (RR 1.020 [95% CI 1.004–1.050]), after which the cumulative RR decreased.

The relationship between relative humidity and HFMD is shown in Fig. 6. When the relative humidity was below 20%, the risk of HFMD increased at lags of 3–7 days and 12–14 days. In addition, relative humidity greater than 80% increased the risk at lags of 3–9 days (Fig. 6c). Figure 6d shows the cumulative exposure response curve of relative humidity and HFMD, which has an approximate “U” shape. The cumulative RR of HFMD first decreased and then increased with increasing relative humidity.

### The association of meteorological factors with HFMD by different levels of social factors

**Fig. 7:**
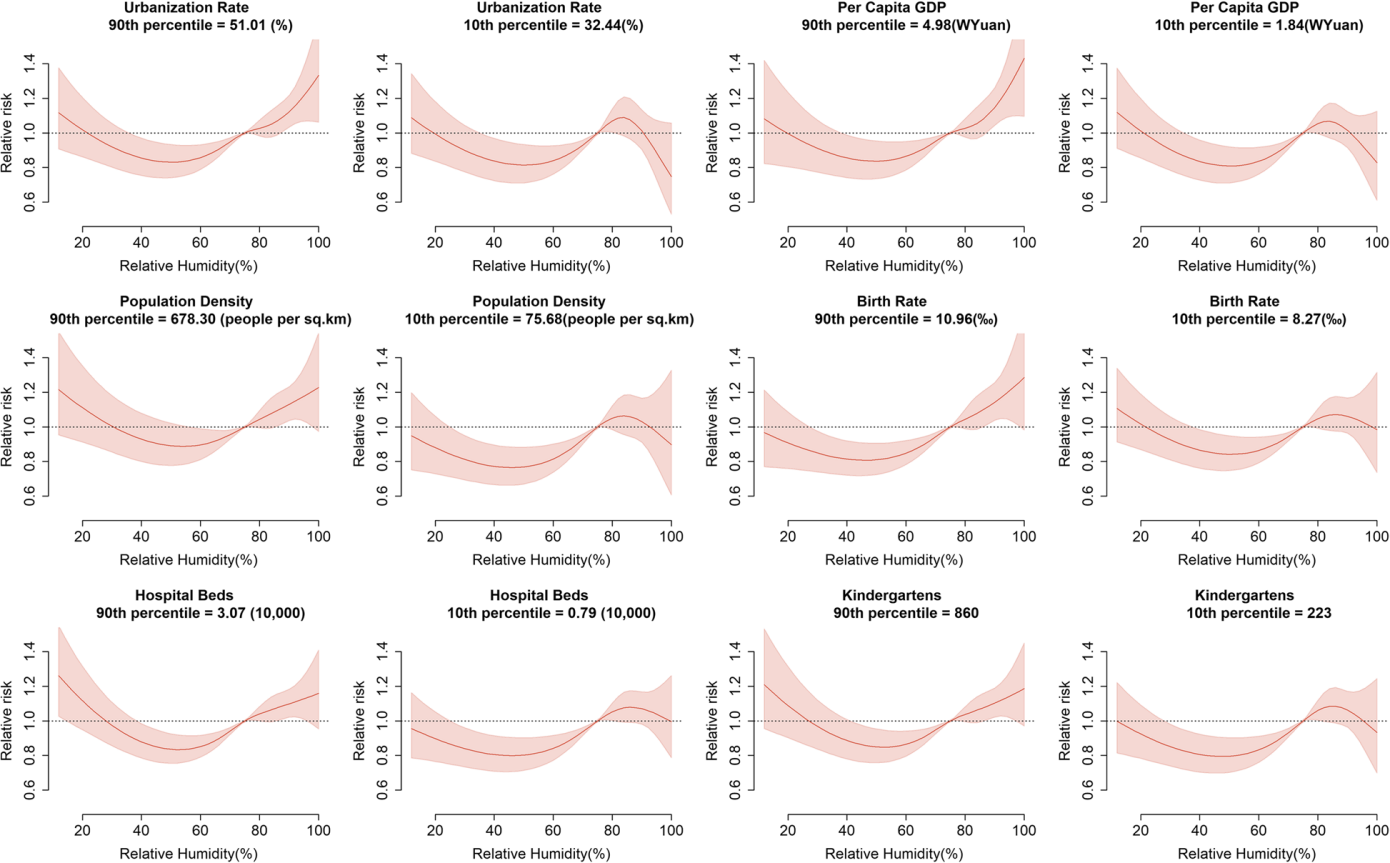
The cumulative RR of relative humidity by level of social factors

According to different percentiles of social factors, the average effect of meteorological factors on HFMD in Sichuan Province was stratified by the gradient of social factors. The results of the cumulative exposure–response curve of relative humidity and HFMD under different levels of social factors are shown in Fig. 7, which contains cumulative exposure responses at high level (90th percentile) and low level (10th percentile) of social factors. Overall, the higher the level of social factors, the higher the cumulative relative risk of relative humidity to HFMD. The correlation curve between relative humidity and HFMD changed from a “U” shape to an “S” shape with the decrease in social factors.

For the urbanization rate, when the relative humidity was greater than 80%, the RR of HFMD in areas with high levels of urbanization and areas with low levels of urbanization had the opposite trend. In highly urbanized areas, the cumulative RR of HFMD increased significantly with increasing relative humidity. In contrast, the cumulative RR of HFMD in areas with low levels of urbanization first increased and then decreased with increasing relative humidity, and the lower the urbanization level was, the greater the decline. Per capita GDP, population density, birth rate, number of beds in health care centers and number of kindergartens were similar to the urbanization rate, with an increasing cumulative RR of high relative humidity under higher levels.

**Fig. 8:**
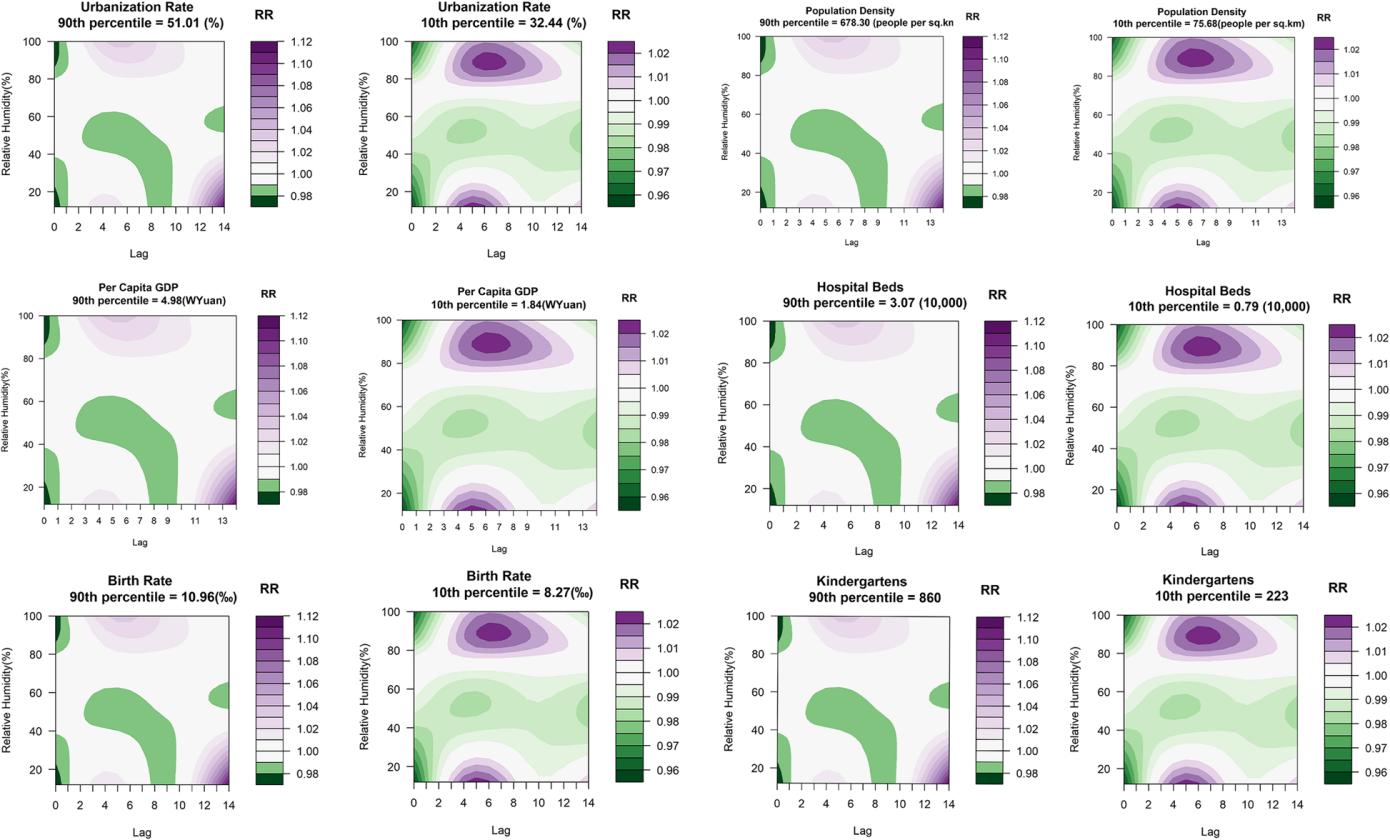
Association between the risk of HFMD and relative humidity at different time lags overall by level of social factors

Figure 8 is a contour map of relative humidity and HFMD at different levels of social factors. For urbanization, on the whole, the risk of HFMD in highly urbanized areas was higher than that in areas with low levels of urbanization. When relative humidity was low (≤ 20%), the risk of HFMD was higher in highly urbanized areas during lag days 3–7; when relative humidity was high (≥ 80%), the risk of HFMD in both regions peaked around lag day 6. The difference is that the RR of HFMD in areas with low levels of urbanization peaked at 86% relative humidity and then gradually decreased with increasing relative humidity, while the risk of HFMD increased in highly urbanized areas. In addition, the lag effect of relative humidity lasted longer in highly urbanized areas. The effects of per capita GDP and birth rate were similar to those of urbanization rate.

For population density, when relative humidity was high (≥ 80%), the risk of HFMD was higher in areas with a low population density on lag days 4–10 than in areas with a high population density. When relative humidity was low (≤ 20%), the risk of HFMD was higher in low population density areas on lag days 4–7 but greater in high population density areas after lag day 10. The effect of the number of beds and kindergartens was similar to that of population density, with a higher risk of HFMD in low-level areas during lag days 4–9 when relative humidity was higher. There was no significant difference in the association between average temperature and HFMD under different levels of social factors.

## Discussion

We used a Bayesian spatiotemporal model combined with a DLNM to analyze the nonlinear lag associations between average temperature, relative humidity and the risk of HFMD in Sichuan Province and to further investigate the influence of social factors on these associations. Relative humidity and average temperature increased the risk of HFMD within a certain range. The urbanization rate, per capita GDP, population density, birth rate, number of beds in health care centers and number of kindergartens interacted with relative humidity. With the increase in the level of social factors, the correlation curve between relative humidity and HFMD changed from an “S” shape to a “U” shape. Social factors did not have a significant influence on the association of average temperature with HFMD.

### The impact of meteorological factors on HFMD

We analyzed the effect of daily average temperature on HFMD. The cumulative relative risk curve for average temperature approximated an inverted "V" shape, and the highest cumulative RR peaked at 19 °C. This result is consistent with the results of Guilin, Wuhan et al. [28, 32, 33]. The relationship between relative humidity and HFMD had an approximate “U” shape. Higher and lower relative humidity increased the risk of HFMD, while the median humidity showed a protective effect. The results are consistent with those in Ningbo, Xiamen and mainland China [21, 34, 35]. The possible mechanisms by which temperature and humidity affect HFMD could be explained as follows. First, the stability, survival time and infectivity of enteroviruses in the external environment could be affected by temperature and humidity [36, 37]. A virology study found that virus survival was proportional to relative humidity when the temperature was within a certain range, and high levels of relative humidity could prolong virus survival on fomites [38], strengthening the transmission of HFMD under favorable meteorological conditions [39]. Second, children are more engaged in outdoor activities during warmer times, which increases the chances of contact with infected individuals or contaminated environments [40]. In addition, children are more susceptible to infection due to reduced sweating in high relative humidity, which leads to decreased metabolism [41]. The above explanations at the virus and host levels may help explain the epidemiology of HFMD resulting from meteorological factors.

### The influence of social factors on the association between relative humidity and HFMD

We found that the relationship between relative humidity and HFMD varied under different levels of social factors. As the level of social factors increased, the correlation curve between relative humidity and HFMD changed from an "S" to an approximate "U" shape, especially the correlation between high relative humidity and HFMD, which changed significantly. The potential mechanism may be related to relative humidity and social factors affecting the biological basis of HFMD epidemics. The source of infection, route of transmission and susceptible population coexist and interact with each other to form the epidemic process of HFMD. When the number of HFMD immune population decrease and susceptible people increase, the greater the susceptibility of the population, and the more conducive it is to the prevalence of HFMD. In addition, the complex transmission routes of HFMD provide multiple ways for pathogens to change hosts and increase the chance of contact with susceptible populations.

The urbanization rate and population density are closely related to the susceptible population and transmission route of HFMD. The results showed that under high relative humidity conditions, the cumulative relative risk of HFMD was higher in areas with high urbanization and high population density, which may be caused by the following two reasons. First, a higher urbanization rate means that more people migrate to cities, including a large number of susceptible people such as children [42], resulting in an increase in the susceptible population of HFMD in areas with a high urbanization rate. At the same time, these areas have developed highways and railways, high population mobility and more frequent contact between people, which is conducive to the spread of HFMD [14]. Second, under high relative humidity conditions, enteroviruses adhere to surfaces more easily and survive longer on fomites [38, 43], thus increasing people’s exposure to viruses. Therefore, on the basis that high relative humidity affects the virus in the environment, frequent contact and the increase in susceptible people are conducive to the spread of HFMD, which in turn strengthens the impact of high relative humidity.

Per capita GDP and the number of beds in health care centers reflect the regional economic level and the availability of regional medical and health resources [27]. In general, areas with a high per capita GDP also have more beds in health care centers. We found that the cumulative relative risk of HFMD was higher in regions with a high per capita GDP under high relative humidity conditions. Bo et al*.* suggested that the effect of health resources on the association of relative humidity with HFMD may be related to the increased number of reported cases [21]. Compared to areas with a lower GDP per capita, people in areas with a higher GDP per capita usually have a higher educational level and pay more attention to HFMD. At the same time, it is easier for people to go to the hospital for treatment in time because of the high availability of medical and health resources, so more HFMD cases are reported [44]. In addition, areas with developed medical resources also have higher population density and population mobility, and for infectious diseases, the impact of demographic factors may be far greater than the impact of medical levels. The underlying mechanism may be as mentioned above, and more research is still needed to clarify this possibility in the future.

An increased birth rate implies more newborns in the whole population, increasing the susceptibility in the general population, which in turn strengthens the effect of relative humidity. In regions with a low level of social factors, the cumulative relative risk of HFMD gradually decreased as the relative humidity increased over approximately 82%. The exact mechanism is still unclear. Other unknown or unmeasured factors may be involved, such as dominant HFMD serotypes. Previous studies found that dominant HFMD serotypes vary between prefecture-level cities in Sichuan Province [9], and the survival time of different pathogen strains is different due to relative humidity [45]. This may cause differences in the survival time of the virus in high relative humidity in various regions, which needs more research to provide relevant information.

### Strengths and limitations

This is the first study to analyze the influence of social factors on the association between meteorological factors and HFMD based on the combination of a Bayesian spatiotemporal model and DLNM. The influence of meteorological factors on HFMD was stratified according to the gradient of social factors, and the temporal and spatial dependence of HFMD was considered by including spatiotemporal random effects. Our findings provide information for evaluating the risk of HFMD in region-specific social and natural conditions. A limitation of our study is that due to the unavailability of data, the social factors analyzed in our study did not include specific local preventive control measures or policy-related indicators, which are very important for the HFMD epidemic. In follow-up studies, we will consider how to quantitatively evaluate preventive control measures in various places and then estimate their impact on HFMD.

## Conclusion

In general, we explored the association of meteorological factors with HFMD in Sichuan Province from 2011 to 2017 and the variations in their associations with HFMD under different levels of social factors. We found that social factors (urbanization rate, per capita GDP, population density, birth rate, number of beds in health institutions, and number of kindergartens) enhanced the influence of high relative humidity on HFMD transmission. The results of our study are helpful for understanding the comprehensive effects of social and meteorological factors on HFMD and provide information for formulating appropriate intervention strategies in regions with different levels of social factors. For example, for areas with a high urbanization rate, such as Chengdu, we should strengthen active surveillance of HFMD during periods of increased relative humidity. At the same time, we can increase HFMD advocacy work for key places such as kindergartens and prepare in advance to prevent a possible new epidemic.

## Supplementary Information

**Additional file 1: Fig S1.** Geographic distribution of weather monitoring stations in Sichuan Province. (The base map is from Resource and Environment Science and Data Center). **Fig S2.** Effect of degrees of freedom to exposure-response relationship on model (a) average temperature; (b) relative humidity. **Fig S3.** Cumulative effects of average temperature and relative humidity on HFMD under different degrees of freedom for exposure response relationship. **Fig S4.** Cumulative effects of average temperature and relative humidity on HFMD under different degrees of freedom for lag response relationship. **Fig S5.** Scatter plot of meteorological factors and HFMD counts. **Fig S6.** Sensitivity analysis of the inclusion form of meteorological confounding factors. **Fig S7.** Temporal changes in the incidence of HFMD and social factors. **Fig S8.** The correlation between HFMD counts and meteorological and social variables in Sichuan Provence from 2011 to 2017. **Fig S9.** Cumulative effects of relative humidity on HFMD under different percentiles of social factors. **Table S1.** Description of daily HFMD counts, meteorological and social variables in 21 prefectures in Sichuan Province.

## Data Availability

The data generated and analyzed during the current study are available from the Sichuan CDC, but restrictions apply to the availability of these data, which were used under license for the current study and are not publicly available. However, data are available from the corresponding author upon reasonable request with permission from the Sichuan CDC.
