# Supplementary material for: Influence of social and meteorological factors on hand, foot, and mouth disease in Sichuan Province
Source: BMC Public Health. 2023 May 10;23:849. doi: 10.1186/s12889-023-15699-4 (PMC10170695; doi:10.1186/s12889-023-15699-4)
Supplement: Supplementary file 1 — Additional file 1: Fig S1. Geographic distribution of weather monitoring stations in Sichuan Province. (The base map is from Resource and Environment Science and Data Center). Fig S2. Effect of degrees of freedom to exposure-response relationship on model (a) average temperature; (b) relative humidity. Fig S3. Cumulative effects of average temperature and relative humidity on HFMD under different degrees of freedom for exposure response relationship. Fig S4. Cumulative effects of average temperature and relative humidity on HFMD under different degrees of freedom for lag response relationship. Fig S5. Scatter plot of meteorological factors and HFMD counts. Fig S6. Sensitivity analysis of the inclusion form of meteorological confounding factors. Fig S7. Temporal changes in the incidence of HFMD and social factors. Fig S8. The correlation between HFMD counts and meteorological and social variables in Sichuan Provence from 2011 to 2017. Fig S9. Cumulative effects of relative humidity on HFMD under different percentiles of social factors. Table S1. Description of daily HFMD counts, meteorological and social variables in 21 prefectures in Sichuan Province. [file 12889_2023_15699_MOESM1_ESM.docx]

**Influence of social and meteorological factors on hand, foot, and mouth disease in Sichuan Province**

**SUPPLEMENTARY FIGURES**

**Fig S1: Geographic distribution of weather monitoring stations in Sichuan Province. (The base map is from Resource and Environment Science and Data Center)**

**Fig S2: Effect of degrees of freedom to exposure-response relationship on model (a) average temperature; (b) relative humidity.**

**Fig S3: Cumulative effects of average temperature and relative humidity on HFMD under different degrees of freedom for exposure response relationship.**

**Fig S4: Cumulative effects of average temperature and relative humidity on HFMD under different degrees of freedom for lag response relationship.**

**Fig S5: Scatter plot of meteorological factors and HFMD counts.**

**Fig S6: Sensitivity analysis of the inclusion form of meteorological confounding factors.**

**Fig S7: Temporal changes in the incidence of HFMD and social factors.**

**Fig S8: The correlation between HFMD counts and meteorological and social variables in Sichuan Provence from 2011 to 2017.**

**Fig S9: Cumulative effects of relative humidity on HFMD under different percentiles of social factors.**

**SUPPLEMENTARY TABLE**

**Table S1: Description of daily HFMD counts, meteorological and social variables in 21 prefectures in Sichuan Province.**


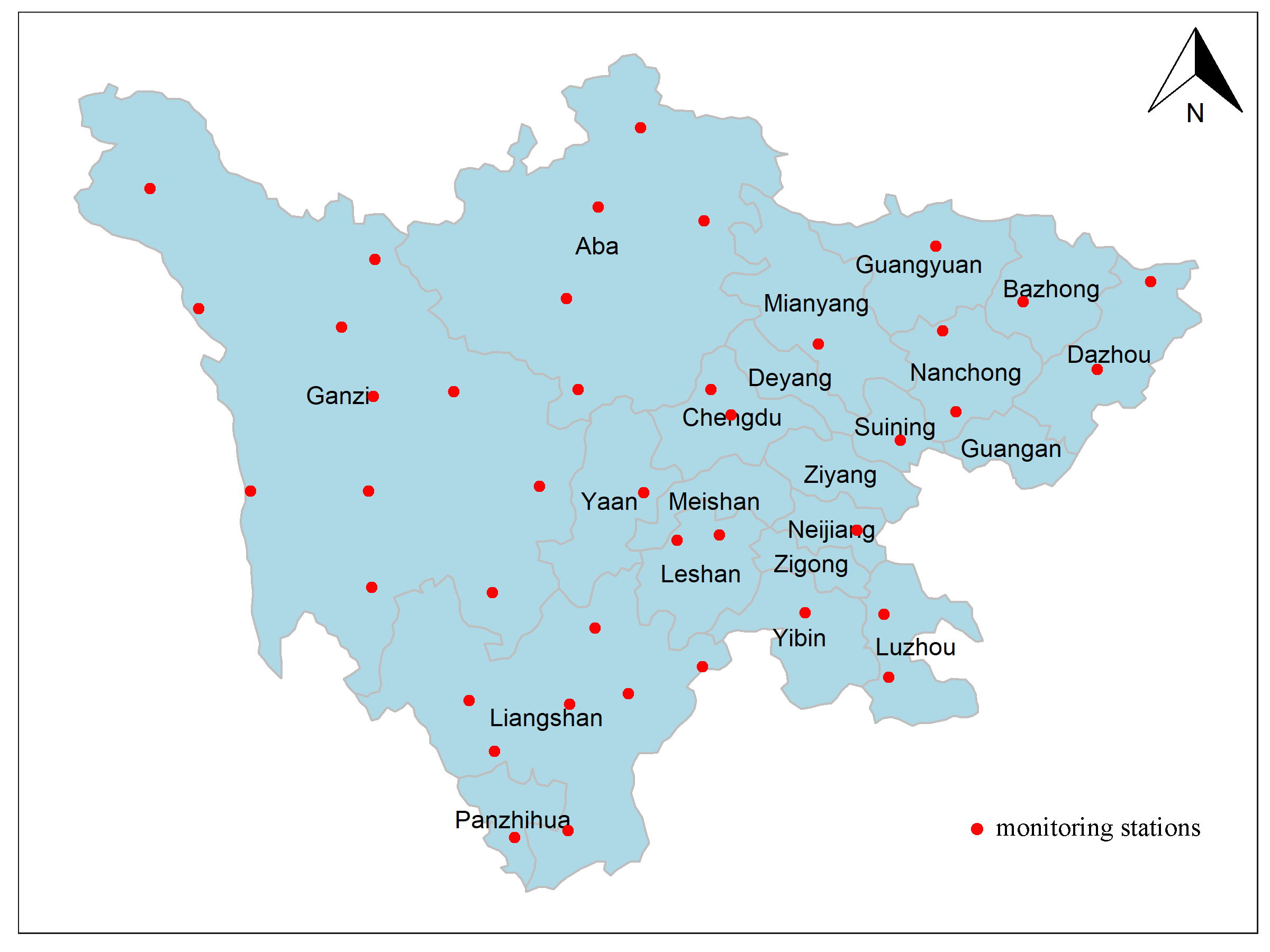


Fig S1. Geographic distribution of weather monitoring stations in Sichuan Province. (The base map is from Resource and Environment Science and Data Center)


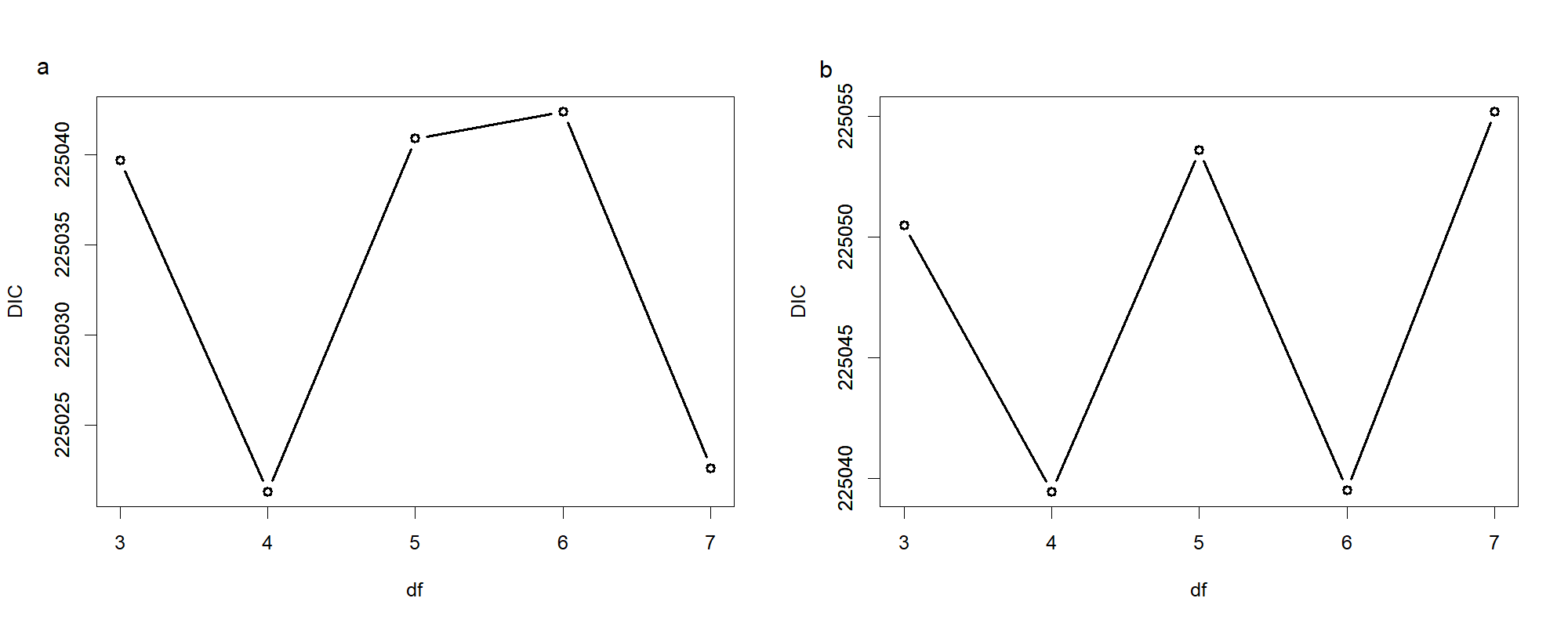
Fig S2. Effect of degrees of freedom to exposure-response relationship on model (a) average temperature; (b) relative humidity.

**
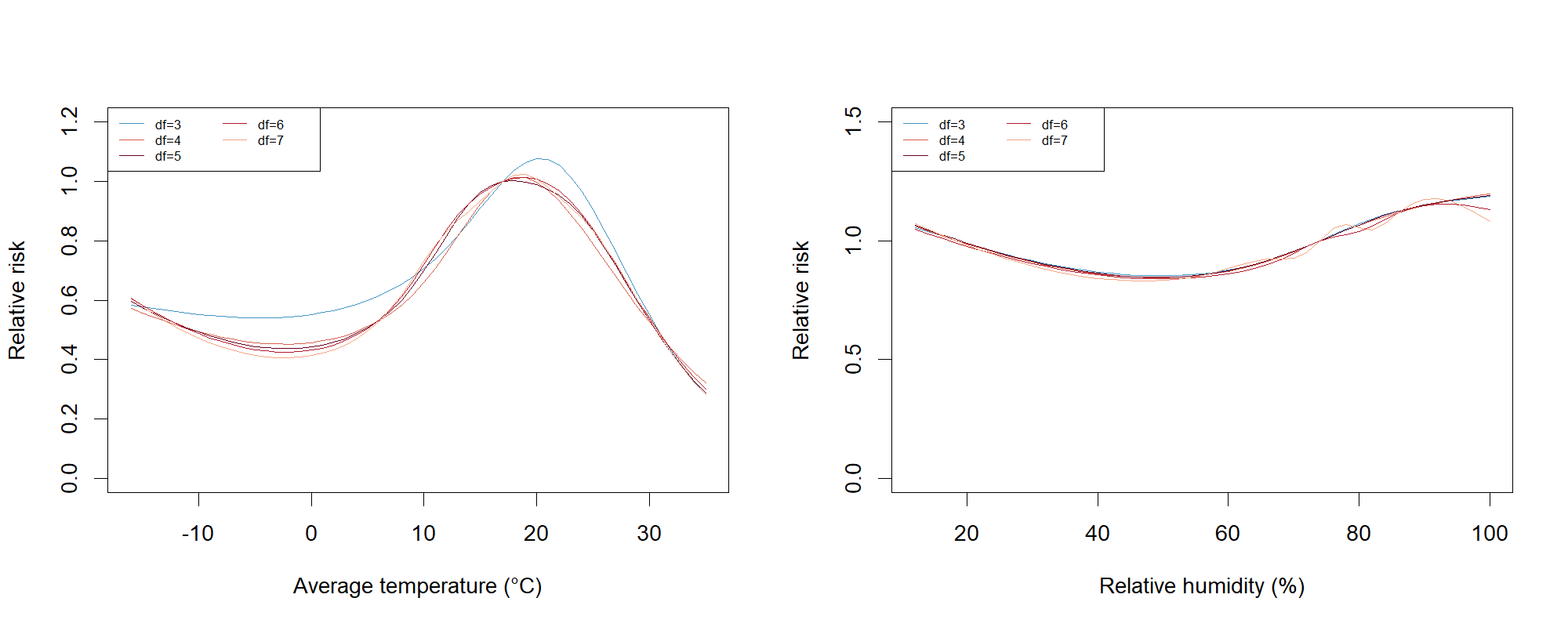
**

Fig S3. Cumulative effects of average temperature and relative humidity on HFMD under different degrees of freedom for exposure response relationship.


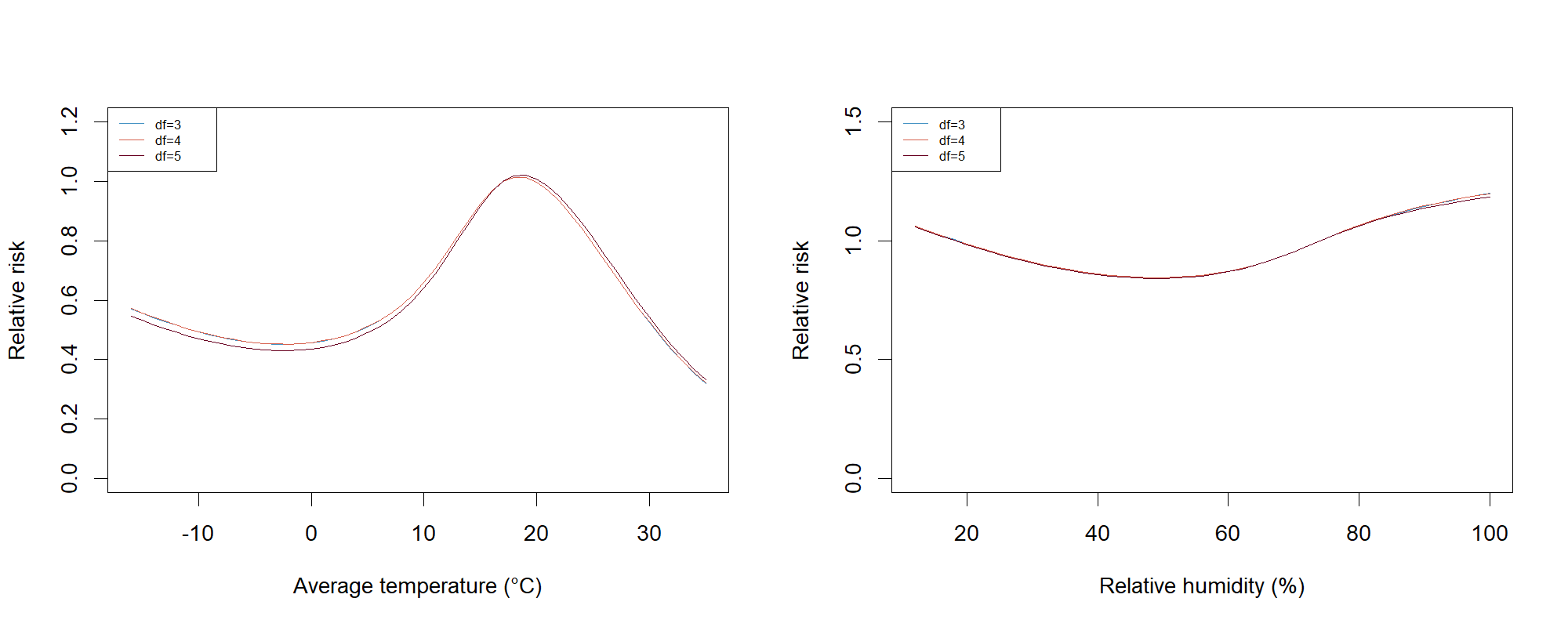


Fig S4. Cumulative effects of average temperature and relative humidity on HFMD under different degrees of freedom for lag response relationship.


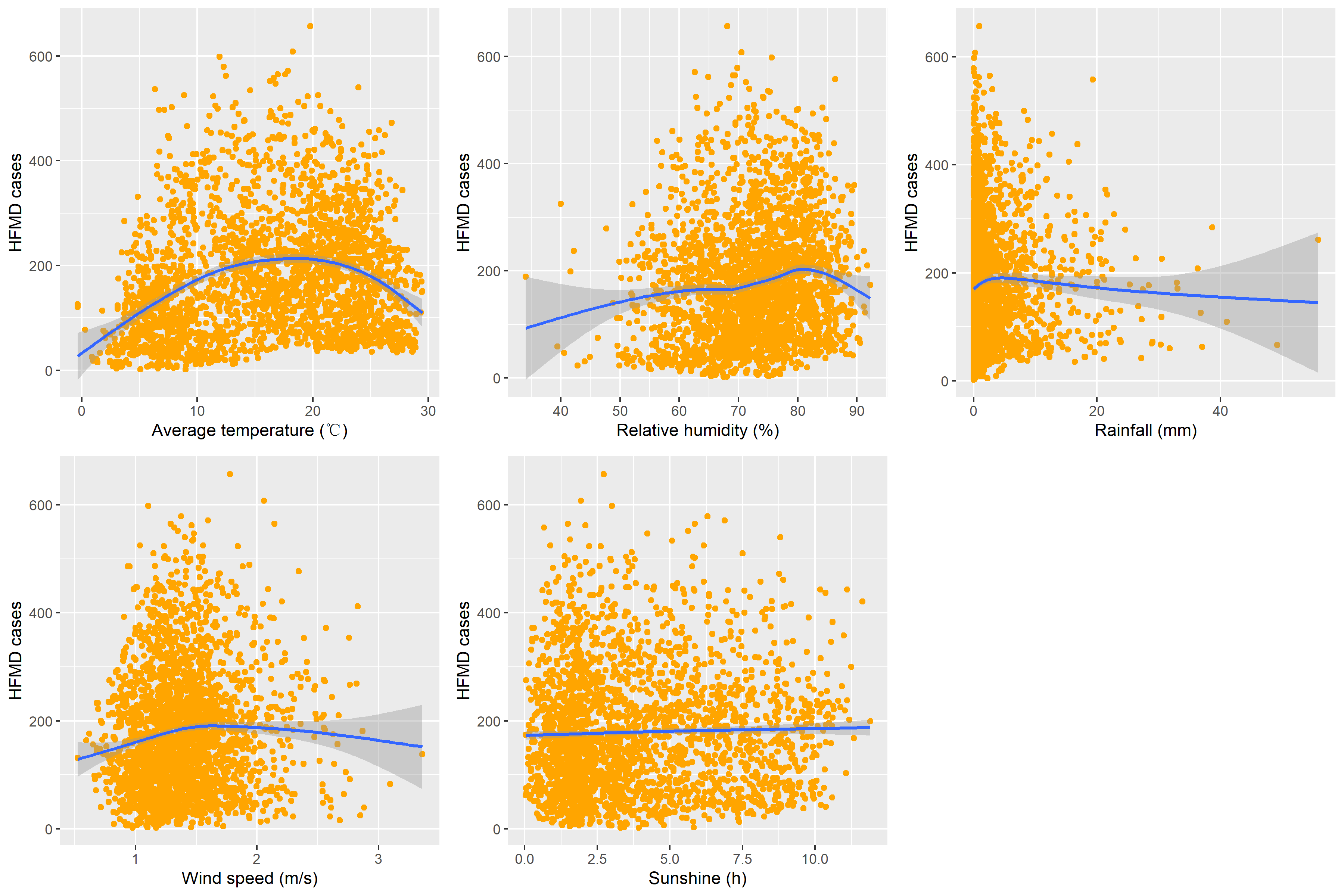


Fig S5. Scatter plot of meteorological factors and HFMD counts.


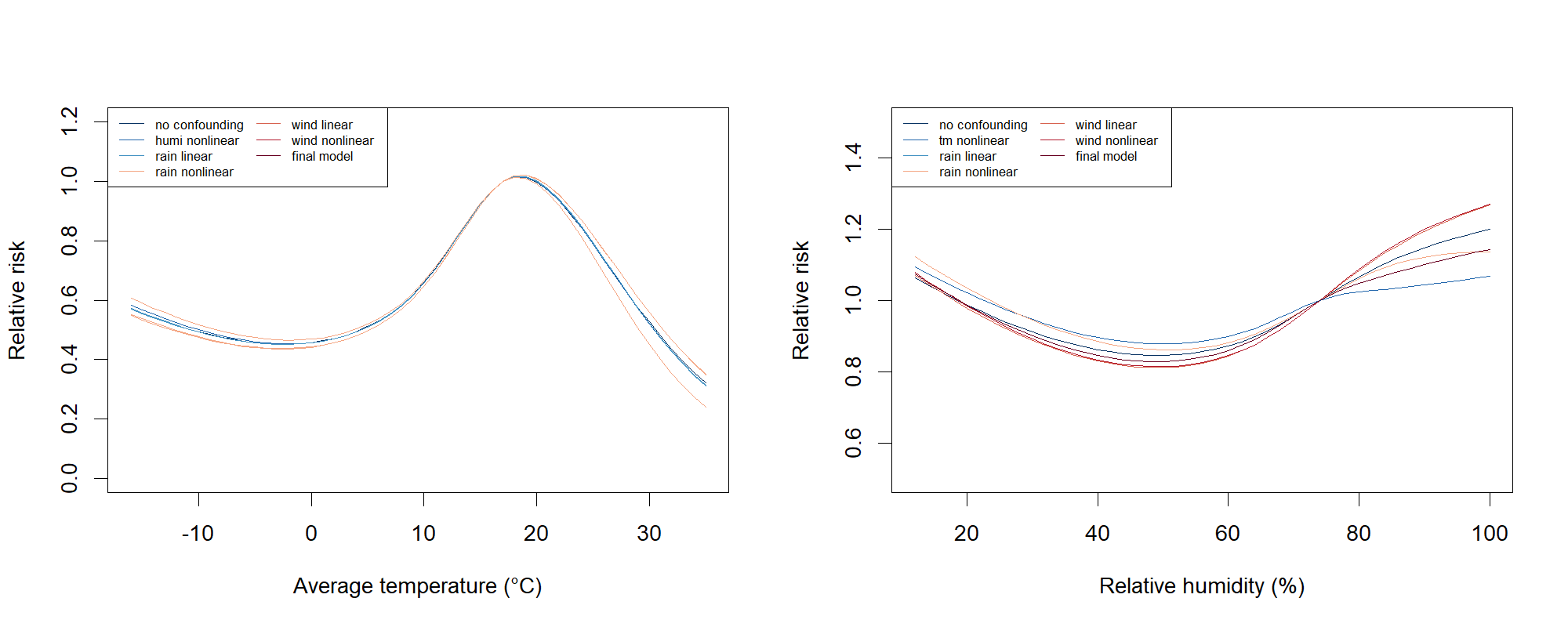


Fig S6. Sensitivity analysis of the inclusion form of meteorological confounding factors.


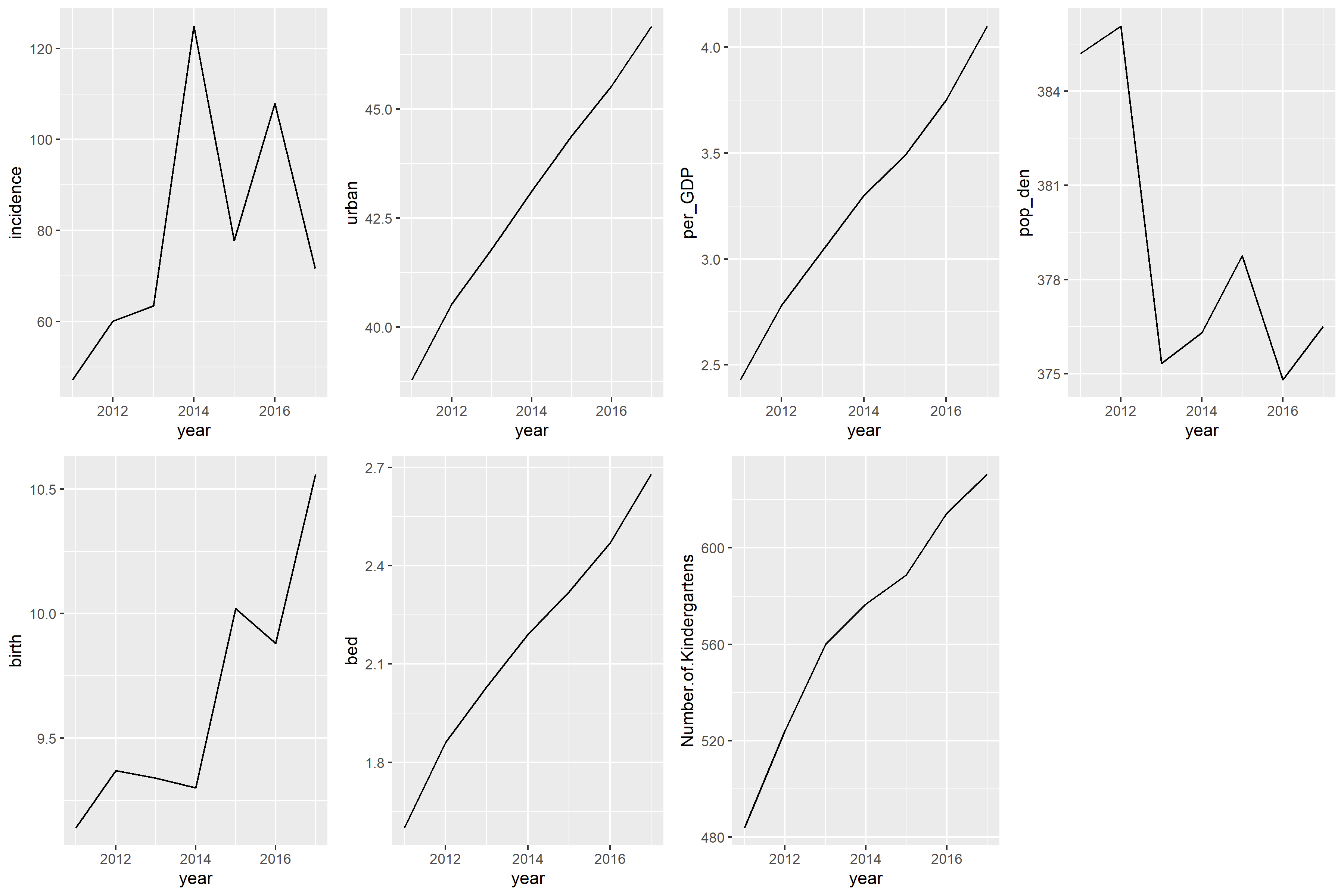
Fig S7. Temporal changes in the incidence of HFMD and social factors.


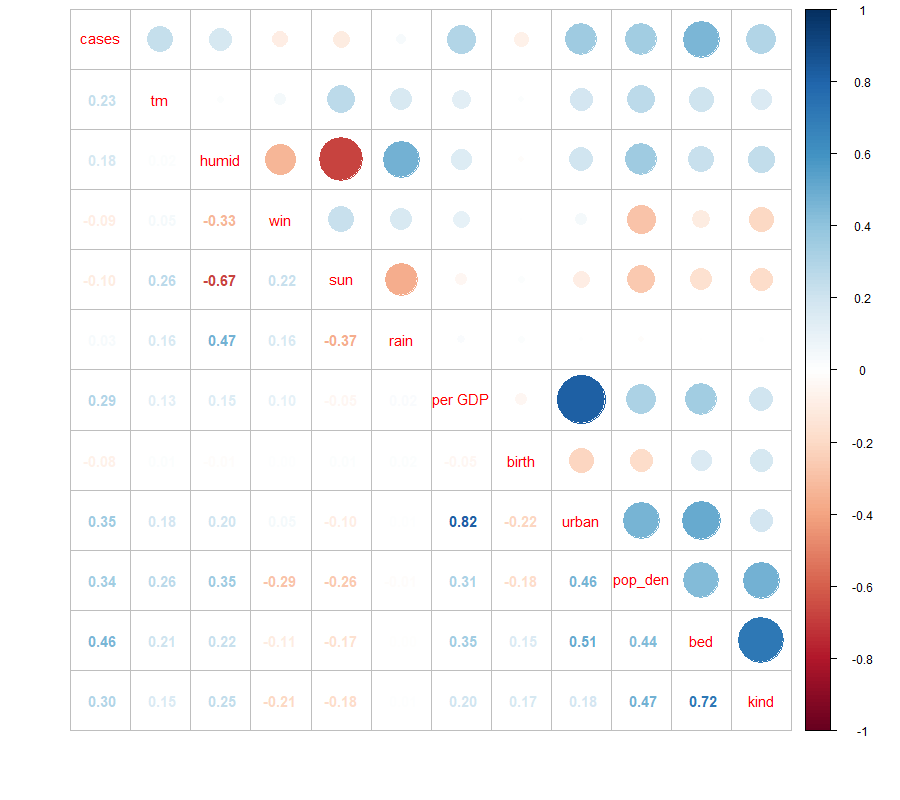


Fig S8. The correlation between HFMD counts and meteorological and social variables in Sichuan Provence from 2011 to 2017.





Fig S9. Cumulative effects of relative humidity on HFMD under different percentiles of social factors.

Table S1. Description of daily HFMD counts, meteorological and social variables in 21 prefectures in Sichuan Province

| city | tm (℃) | humid (%) | Per GDP (WYuan) | Urban (%) | Pop den (per sq.km ) | Birth Rate (‰) | Beds (10,000) | Kindergartens |
| --- | --- | --- | --- | --- | --- | --- | --- | --- |
| Chengdu | 17.3(6.7, 26.8) | 76.7(63.0, 88.8) | 6.8(4.9, 8.7) | 69.9(67.0, 71.8) | 1166.1(1110, 1209.5) | 9.3(7.1, 12.6) | 10.8(8.0, 13.5) | 1970(1699, 2366) |
| Zigong | 18.4(7.9, 28.1) | 79.7(65.6, 91.9) | 3.9(2.9, 4.6) | 46.7(42.7, 50.9) | 647.2(625.0, 678.3) | 9.5(8.6, 10.3) | 1.6(1.2, 2.0) | 388(337, 427) |
| Panzhihua | 17.2(9.7, 23.3) | 58.1(31.9, 78.4) | 7.1(5.3, 9.3) | 64.0(61.6, 66.0) | 169.2(166.5, 175.8) | 8.6(7.4, 9.9) | 0.9(0.8, 1.0) | 199(188, 210) |
| Luzhou | 18.4(8.1, 28.1) | 82.3(66.8, 95.8) | 2.9(2.1, 3.7) | 44.6(39.9, 49.0) | 350.8(347.0, 354.2) | 10.3(9.4, 11) | 2.3(1.5, 2.9) | 623(469, 727) |
| Deyang | 17.6(7.1, 27.2) | 72.3(56.0, 86.9) | 4.3(3.2, 5.6) | 47.1(43.0, 51.0) | 595.1(588.5, 598.6) | 9.3(8.8, 11.2) | 1.9(1.6, 2.3) | 266(228, 325) |
| Mianyang | 15.5(5.1, 24.8) | 69.0(54.0, 83.3) | 3.4(2.6, 4.3) | 46.5(41.8, 51.0) | 234.4(231.0, 239.0) | 8.8(7.8, 10.0) | 3.0(2.2, 3.6) | 621(510, 744) |
| Guangyuan | 16.6(5.8, 26.5) | 69.0(51.9, 85.3) | 2.2(1.6, 2.8) | 39.3(34.7, 44.0) | 159.1(155.6, 163.0) | 9.5(8.5, 10.4) | 1.7(1.2, 2.1) | 281(250, 300) |
| Suining | 17.9(7.3, 28.0) | 76.6(60.3, 91.1) | 2.6(1.9, 3.5) | 44.4(40.0, 48.5) | 626.2(608.0, 653.5) | 8.3(7.8, 9.0) | 1.6(1.1, 2.0) | 397(279, 489) |
| Neijiang | 18.2(7.7, 28.0) | 79.7(65.6, 92.2) | 3.0(2.3, 3.6) | 44.2(40.2, 47.9) | 708.3(692.0, 743.6) | 9.3(8.1, 12.6) | 1.9(1.4, 2.3) | 611(500, 675) |
| Leshan | 16.4(6.3, 25.7) | 78.3(64.6, 90.3) | 3.7(2.8, 4.6) | 45.8(41.2, 50.2) | 254.5(249.5, 257.0) | 8.9(7.4, 10.9) | 1.8(1.5, 2.2) | 529(413, 698) |
| Nanchong | 17.8(7.1, 28.1) | 75.6(61.1, 89.2) | 2.2(1.6, 2.9) | 42.2(37.5, 46.5) | 514.2(506.0, 525.0) | 8.7(8.4, 9.2) | 3.0(2.2, 4.0) | 752(532, 854) |
| Meishan | 18.4(8.0, 27.8) | 78.6(65.7, 90.0) | 3.2(2.3, 4.0) | 40.4(35.8, 44.8) | 419.9(417.0, 423.8) | 10.5(9.2, 11.5) | 1.5(1.0, 1.9) | 452(437, 466) |
| Yibin | 18.7(8.3, 28.3) | 77.4(62.3, 90.7) | 3.2(2.4, 4.1) | 43.8(39.4, 48.1) | 340.1(337.0, 343.1) | 10.5(10.3, 10.8) | 2.5(1.8, 3.2) | 828(784, 889) |
| Guangan | 18.3(7.6, 28.8) | 76.6(59.7, 91.5) | 2.8(2.1, 3.6) | 35.7(30.9, 40.2) | 518.5(509.0, 536.1) | 9.4(8.9, 10.1) | 1.4(1.0, 1.8) | 714(684, 736) |
| Dazhou | 17.3(6.6, 27.7) | 72.7(59.3, 86.3) | 2.4(1.8, 2.8) | 39.3(34.3, 43.9) | 338.3(332.0, 343.3) | 10.1(9.8, 10.5) | 2.3(1.7, 3.0) | 725(681, 752) |
| Yaan | 14.6(4.8, 23.5) | 75.1(63.2, 86.0) | 3.1(2.3, 3.9) | 41.1(36.6, 45.4) | 102.0(101.1, 102.8) | 9.0(8.5, 10.2) | 1.0(0.8, 1.2) | 236 (218, 253) |
| Bazhong | 16.4(5.6, 26.7) | 71.1(57.9, 84.0) | 1.4(1.0, 1.8) | 36.1(31.3, 40.5) | 271.5(269.0, 275.7) | 9.7(8.8, 10.4) | 1.5(1.0, 1.9) | 222(194, 259) |
| Ziyang | 18.0(7.5, 27.9) | 77.9(62.3, 91.4) | 3.2(2.3, 4.0) | 38.1(34.4, 41.3) | 447.5(442.0, 453.8) | 9.8(8.5, 11.0) | 1.9(1.5, 2.2) | 1067(849, 1195) |
| Aba | 3.7( -6.5, 12.8) | 65.5(46.7, 81.3) | 2.6(1.9, 3.1) | 35.6(31.6, 38.9) | 11.0(10.9, 11.2) | 8.9(8.4, 9.9) | 0.4(0.3, 0.5) | 243(61, 344) |
| Ganzi | 8.1( -0.9, 16.3) | 51.4(29.5, 73.4) | 1.8(1.4, 2.2) | 26.8(22.4, 30.6) | 7.7(7.2, 8.0) | 10.4(9.5, 11.2) | 0.4(0.3, 0.5) | 362(331, 396) |
| Liangshan | 15.5(7.4, 22.5) | 61.7(38.8, 83.3) | 2.7(2.2, 3.1) | 31.4(28.2, 34.3) | 77.4(75.7, 80.0) | 14.2(10.9, 19.3) | 2.0(1.3, 2.6) | 411(247, 573) |
